# Aqueous extracts of *Elsholtzia ciliata* and *Hovenia dulcis* ameliorate loperamide-induced constipation in mice by promoting intestinal peristalsis and barrier function and the abundance of intestinal beneficial bacteria

**DOI:** 10.3389/fmicb.2025.1531232

**Published:** 2025-05-13

**Authors:** Junnan Wu, Qilei Xin, Shuo Wang, Xu Zhang, Chunping Jiang

**Affiliations:** ^1^Nanjing Drum Tower Hospital Clinical College of Nanjing University of Chinese Medicine, Nanjing, China; ^2^Department of Hepatobiliary Surgery, Affiliated Drum Tower Hospital of Nanjing University Medical School, Nanjing, China; ^3^Jinan Microecological Biomedicine Shandong Laboratory, Jinan, Shandong, China

**Keywords:** constipation, *Elsholtzia ciliata*, *Hovenia dulcis*, gut microbiota, loperamide, edible traditional Chinese medicine (ETCM)

## Abstract

**Objective:**

The aims of the present study were to determine the efficacy of edible traditional Chinese medicines (ETCMs) in treating constipation, verify their laxative effects, and conduct preliminary investigations into their mechanisms of action.

**Methods:**

ICR mice were treated with loperamide to induce constipation, and various fecal parameters, including fecal volume, water content, and intestinal transport function, were measured in these constipation model mice to screen for ETCMs with laxative properties. The mechanism of action was preliminarily explored by examining changes in the intestinal mucosal structure, protein expression levels, and alterations in intestinal flora composition.

**Results:**

In ICR mice with loperamide-induced constipation, *Elsholtzia ciliata* aqueous extract (ECAE) and *Hovenia dulcis* aqueous extract (HDAE) significantly ameliorated constipation symptoms, mitigated colonic pathological tissue damage, significantly increased the expression levels of proteins associated with the promotion of intestinal peristalsis [Stem Cell Factor Receptor (c-Kit) and Stem Cell Factor (SCF)] and the maintenance of the intestinal barrier [Zonula Occludens-1 (ZO-l), Occludin and Claudin-l], and promoted beneficial intestinal bacterial colonization.

**Conclusion:**

ECAE and HDAE ameliorated constipation in mice, and their mechanism of action may be related to the increased abundance of intestinal bacteria such as *Turicibacter, Olsenella*, and *Odoribacter*, which contribute to higher butyrate production. This increase in butyric acid reduces inflammation, improves intestinal barrier function, and increases the abundance of beneficial intestinal bacteria.

## 1 Introduction

Constipation is a common functional gastrointestinal disorder with symptoms including hard dry stools, inability to pass stools and incomplete bowel movements (Bharucha and Lacy, 2020). The appearance of these symptoms is often closely related to abnormal intestinal function. Studies have shown that the occurrence of constipation may be related to enteric nervous system dysfunction, visceral allergic reactions, abnormal distribution of interstitial Cajal cells and changes in gastrointestinal regulatory peptide levels (King et al., 2010). Because of the difficulty of defecation, constipation patients often experience a loss of appetite, stomach pain, heartburn, acid reflux, heartburn, nausea, vomiting and other gastrointestinal symptoms (Varni et al., 2015). These symptoms not only affect the patient's daily diet and sleep but also cause mood swings and increase psychological stress. Moreover, long-term constipation may also lead to complications such as toxemia, metabolic disorders and even neurasthenia, posing a threat to the physical and mental health of patients (Sumida et al., 2019). The medical community has developed various methods for constipation treatment. Medications represent one of the most used methods. For example, stool softeners can increase stool moisture for easier discharge; osmotic laxatives increase the intestinal osmotic pressure and stimulate intestinal peristalsis by absorbing intestinal water; and stimulant laxatives can directly stimulate the intestinal mucosa and intestinal wall nerve plexus and promote intestinal propulsive peristalsis (Bharucha and Wald, 2019). However, although these drugs can relieve symptoms, they are also associated with side effects such as diarrhea, nausea, abdominal pain and headache, and long-term use can lead to drug dependence and high recurrence rates (Chang et al., 2014). Therefore, there is an urgent need to discover drugs that can safely treat constipation without side effects; ideally, these drugs could be incorporated into daily diets.

Lifestyle, diet, sex, regional differences, and drugs all affect the host's gut flora (Ohkusa et al., 2019; Fan et al., 2022). There are many reports about changes in the intestinal flora during constipation and the recovery of the intestinal flora after treatment (Zhou et al., 2022; Wang R. et al., 2023). The intestinal flora structure of patients with constipation is often disordered (Hofman et al., 2022). Studies have shown that traditional Chinese medicines (TCMs) can effectively relieve constipation by regulating the intestinal microecology, increasing the number of beneficial intestinal bacteria and inhibiting the growth of harmful bacteria (Gao et al., 2021; Zhang et al., 2023). In mechanistic studies, TCMs may indirectly affect the composition and function of the intestinal flora by regulating the inflammatory response, improving intestinal barrier function, and regulating neuroendocrine function (Zhan et al., 2022). The application of 16S rRNA high-throughput sequencing has also facilitated the study of the gut microbiota. The authors employed 16S rRNA sequencing to compare the changes in the intestinal flora before and after modeling in mice (Zhan et al., 2022). The aim of this approach was to investigate the proportion of beneficial bacteria regulated by TCMs that may alleviate constipation.

The pathophysiological mechanisms underlying chronic constipation in humans are highly complex, encompassing not only aberrant colonic motor function, such as increased non-propulsive contractions and prolonged transit time, but also including abnormalities in rectal and anal sphincter function, as well as diminished contractile function of the pelvic floor muscles (Scott et al., 2021; Kilgore and Khlevner, 2024). These factors collectively contribute to difficulties in normal defecation. Loperamide is a commonly used symptomatic drug for the treatment of diarrhea; loperamide mainly delays intestinal peristalsis through multiple mechanisms, such as activating opioid receptors, inhibiting intestinal secretion, increasing endogenous analgesia, and affecting neuroregulation, to reduce the frequency of intestinal emptying (Hughes et al., 1984). Although there are notable differences in the pathophysiology between loperamide-induced constipation and human chronic idiopathic constipation, the clinical manifestations of loperamide-induced constipation are comparable, characterized by reduced bowel movement frequency and quantity, as well as decreased intestinal propulsion rate. Therefore, loperamide hydrochloride was used to establish a constipation model in this study.

According to the information released by China's State Administration for Market Regulation, a total of 110 TCMs have been confirmed to be edible traditional Chinese medicines (ETCMs), which undoubtedly opens up a new way for the application of TCM in modern society. ETCM refers to certain foods and medications that exhibit similar or identical therapeutic effects; thus, health care objectives and disease treatment could be achieved through the consumption of these items in the diet (He et al., 2024). In recent years, ETCMs have gained widespread attention for their unique health and therapeutic effects, which prompted the authors to choose those to regulate constipation through diet. According to ancient Chinese herbal medicine texts such as the herbaceous works of past dynasties as well as modern Chinese Pharmacopeia records, ETCMs such as *Illicium verum, Zoacys dhumnades, Citrus aurantium, Ginkgo biloba, Elsholtzia ciliata, Hovenia dulcis, Agkistrodon halys*, and *Lonicera hypoglauca* have shown promising results in treating illnesses unrelated to gastrointestinal function. However, after extensive literature research and studies, no conclusive findings on the laxative effect of these ETCMs have been found.

In this study, by establishing a loperamide-induced constipation model in ICR mice, two ETCMs with potential laxative effects, *E. ciliata* and *H. dulcis*, were identified. A preliminary mechanistic study of *E. ciliata* aqueous extract (ECAE) and *H. dulcis* aqueous extract (HDAE) was subsequently conducted to provide a theoretical and experimental basis for the development and utilization of ETCMs.

## 2 Materials and methods

### 2.1 Materials

#### 2.1.1 Animals experiments and ethics

Female ICR mice, body mass 20–25 g, 6 weeks old, purchased from Yangzhou University Center for Comparative Medicine, Laboratory Animal Production License No.: SCXK2022-0009. Animal experiments were approved by the Animal Ethical and Welfare Committee of NJU (SYXK2019-0056). Housed in isolation cages, free to feed and drink, and started experiments after 3 days of acclimatization rearing.

#### 2.1.2 Reagents and instruments

The *E. ciliata* and *H. dulcis* were purchased from Bozhou Jingwan Traditional Chinese Medicine Drinking Tablets Factory, China. Loperamide hydrochloride (Manufacture batch number: L129465), Activated charcoal (Manufacture batch number: C112241), Gum arabic (Manufacture batch number: A108975) were purchased from Shanghai Aladdin Biochemical Science and Technology Co., Ltd, China. ZO-1 Rabbit Polyclonal Antibody, Occludin Rabbit Polyclonal Antibody, Claudin-1 Rabbit Polyclonal Antibody (21773-1-AP, 13409-1-AP, 13050-1-AP), Beta Actin Monoclonal antibody (66009-1-Ig) were purchased from Wuhan Sanying Biotechnology Co., Ltd, China. c-Kit antibody (Bs-10005R) and SCF antibody (Bs-0545R) were purchased from Beijing Biosynthesis Biotechnology Co., Ltd, China. Anti-rabbit IgG, HRP-linked Antibody was purchased from CST (#7074). Citrate repair solution (Manufacture batch number: 09172311), Sheep serum for containment (Manufacture batch number: 10022315), HE staining solution (Manufacture batch number: G1004-100ML) were purchased from Wuhan Servicebio Technology Co., Ltd, China.

### 2.2 Methods

#### 2.2.1 Extraction of herbs

First and foremost, in accordance with the traditional decoction method, we meticulously extracted and concentrated each Chinese medicinal material to ensure that the levels of its active ingredients met the experimental requirements. One-hundred gram of each of the Chinese herbs *I. verum, Z. dhumnades, C. aurantium, G. biloba, E. ciliata, H. dulcis, A. halys, L. hypoglauca* were weighed, 10 times the amount of distilled water was added, and the decoction was immersed for 30 min, and then boiled for 2 h, and then 8 times the amount of distilled water was added to the decoction and boiled for 1 h, and then filtered out the medicinal liquid with gauze, and then the medicinal liquids were combined, heated, and then concentrated to obtain the concentrations of raw medicines of 1.43 g·mL^−1^, 1 g·mL^−1^, 1.32 g·mL^−1^, 1 g·mL^−1^, 1.11 g·mL^−1^, 1.11 g·mL^−1^, 1 g·mL^−1^, 0.91 g·mL^−1^, all of which were placed in centrifuge tubes for spare use.

To quantify the dry matter components of the Chinese herbs, 1 mL of each decoction was weighed and dried in an oven at 65°C until constant weight was achieved (with intervals exceeding 3 h, and a weight difference of < 0.3 mg after two consecutive dryings). The concentrations of the decoctions were calculated as 0.158 g·mL^−1^, 0.133 g·mL^−1^, 0.180 g·mL^−1^, 0.154 g·mL^−1^, 0.136 g·mL^−1^, 0.163 g·mL^−1^, 0.147 g·mL^−1^, 0.151 g·mL^−1^, respectively (based on the mass of the dried substance per milliliter of concentrated liquid).

#### 2.2.2 Constipation modeling and intervention

Constructed constipation models were performed using loperamide hydrochloride (hereafter referred to as Lop) based on the results of literature surveys (Kim et al., 2019; Yao et al., 2022; Kim et al., 2023; Park et al., 2023; Tuohongerbieke et al., 2024) and pre-experimental results. ICR mice were randomly divided into a normal control group (NC) and a constipation group, and ICR mice in the loperamide-induced constipation group were further divided into a model group (Lop + saline, Lop), *I. verum* group (Lop + *I. verum*, Iv), *Z. dhumnades* group (Lop + *Z. dhumnades*, Zd), *C. aurantium* group (Lop + *C. aurantium*, Ca), *G. biloba* group (Lop + *G. biloba*, Gb), *E. ciliata* group (Lop + *E. ciliata*, Ec), *H. dulcis* group (Lop + *H. dulcis*, Hd), *A. halys* group (Lop + *A. halys*, Ah), *L. hypoglauca* group (Lop + *L. hypoglauca*, Lh). The ICR mice in the constipation group were injected subcutaneously with 4 mg·kg^−1^ of Lop twice a day for 4 days, and after a 3-day immobilization period, 8 mg·kg^−1^ of Lop was injected subcutaneously for 4 days. The fecal pellet morphology and mental activity status of mice in each group were observed. When the rats showed the following symptoms, decreased activity, decreased water content of fecal pellets, hard texture of feces, and decreased volume of fecal pellets, indicating successful modeling. After constipation induction, the 8 treatment groups were gavaged with the same 8 g·kg^−1^ of aqueous extract of ETCM, while the normal and model groups were given an equal volume of saline by gavage once a day for 7 consecutive days. Standard laboratory food, water and clean cages were provided to all mice.

### 2.3 Measurement of constipation indicators

The general physiological conditions and fecal conditions of mice in each group were observed daily. On the 7th and 14th days of the experiment, fresh feces were collected from each group of mice for 3 h, the number of pellets was recorded, weighed (wet weight = total weight M1-empty dish M0), and then dried in a dryer at 80°C for 6 h. The feces were again weighed (dry weight = total weight M2-empty dish M0), and the water content of the feces was calculated and statistically analyzed, in order to verify the success of the modeling and the therapeutic efficacy of citrulline. After the last administration, the mice were fasted for 16 h. The mice in each group were kept in a single cage and given 0.2 mL of 10% activated charcoal solution by gavage, and the time was started at the end of the gavage. The time of the first black feces and the number of black feces in the mice within 6 h were recorded and statistically analyzed.


$$\begin{array}{l} \;Water\;content\;of\;mouse\;feces\;\left(W\right)\;\\ =\left(M1-M2\right)/\left(M1-M0\right)\;x\;100\% \end{array}$$

### 2.4 Measurement of small bowel propulsion rate

After 16 h of fasting without water at the end of the constipation index measurement, mice in each group were given 0.2 mL of 10% activated charcoal solution by gavage. The time was counted from the end of the gavage, and the mice were immediately executed after 20 min. The whole intestine from the pylorus to the cecum was removed by dissection, slowly straightened and placed on a white paper for photographing, and then the total length of the intestine (L1) and the propulsion distance of 10% activated charcoal solution from the pylorus to the cecum (L2) were measured, and the propulsion rate of the small intestine (R) was calculated and statistically analyzed.


$$\begin{array}{l} \;Small\;bowel\;propulsion\;rate\;\left(R\right)\\ \;=L2/L1\;x\;100\% \end{array}$$

### 2.5 Fecal tissue and colon tissue collection

On the 14th day of the experiment, fecal tissues were collected from each mouse, collected in Eppendorf tubes for liquid nitrogen snap freezing and frozen in −80°C refrigerator for further testing. On the 16th day of the experiment, after the mice were executed by cervical dislocation, the mouse colon tissues were dissected and separated into two parts, one part was sheared, lysed, and milled in preparation for the extraction of total tissue protein, and the other part was fixed in 4% paraformaldehyde for further pathology experiments.

### 2.6 H&E staining

After paraffin-embedded treatment, the mouse colon tissue was cut into 5 μm—thick paraffin thin slices. The slices were taken into environmentally friendly dewaxing solution, anhydrous ethanol and 75% alcohol for dewaxing and washing, and after repeating the water, the slices were washed and stained with 1% hematoxylin and eosin water (H&E) at 25 ± 2°C, and the slices were blocked with neutral gum. Finally, the morphological changes of the colonic tissues were observed under a light microscope and images were acquired.

### 2.7 Immunohistochemistry

The paraffin sections of mouse colon were baked in a desiccator at 65°C for 30 min, then dewaxed, dehydrated and washed. In order to improve the specificity and stability of immunohistochemical staining, 2,000 mL of citrate repair solution with a pH of 6.0 was prepared for antigen repair and thus improve the binding efficiency of antigen and antibody, followed by the addition of goat serum for closure. The goat serum was discarded, c-Kit antibody (1:300) and SCF antibody (1:300) were added dropwise, and the sections were incubated for 1 h. The sections were removed and rinsed with PBS-T. The excess liquid on the sections was discarded. Shake off the excess liquid on the sections, add HRP-labeled goat anti-rabbit IgG antibody dropwise, incubate for 30 min, rinse with PBS-T. The sections were treated with DAB solution for color development for 5 min and hematoxylin re-staining for 1 min. The slices were sealed with neutral gum and observed under a light microscope.

### 2.8 Western blot analysis

Mouse colon tissue was taken, lysate was added and lysed by grinding on ice until homogenized. The supernatant was collected after centrifugation, and it was quantified for protein and adjusted for concentration. A 20 μg protein sample was taken from each well, denatured by boiling and subjected to SDS-PAGE electrophoresis. Then the membrane was immediately transferred by wet transfer method, closed with 5% skimmed milk powder for 1 h. The membrane was washed with TBS-T, followed by the addition of primary antibody (ZO-1 1:5 000, Occludin 1:2 000, Claudin-1 1:1 000) and the internal reference (β-actin 1:60 000) according to molecular weight sizes of the proteins, respectively, and incubated overnight. On the following day, after washing the membrane with TBS-T, HRP-labeled goat anti-rabbit IgG secondary antibody was added and incubated for 1 h. After washing the membrane with TBS-T, protein bands were developed.

### 2.9 16s rRNA gene sequencing

Total DNA was extracted and analyzed from the fecal samples of four mice per group. The V3-V4 hypervariable region of the 16S rRNA gene was amplified using the Illumina MiSeq PE300 platform with the forward primer 338F (ACTCCTACGGGAGGCAGCAG) and reverse primer 806R (GGACTACHVGGGTWTCTAAT). Raw sequencing reads were obtained, followed by quality control, read assembly, and chimera removal. Subsequently, amplicon sequence variants (ASVs) were identified and taxonomic classification was performed on the processed data. Microbial classification macrogenome sequencing 16S rRNA gene sequencing was done by Shanghai Meiji Bio-pharmaceutical Technology Co. (Contract No.: MJ20240612154). All data analysis was performed on the Meiji Bio Cloud platform (https://cloud.majorbio.com).

### 2.10 Statistical analysis

All measurement data were analyzed using GraphPad Prism 9.3.0 statistical analysis software. Comparisons between groups were made by *t*-test, and comparisons between multiple groups were made by one-way ANOVA, with *P* < 0.05 indicating that the differences between experimental groups were statistically significant.

## 3 Results

### 3.1 ECAE and HDAE alleviate constipation in model mice

**Figure 1:**
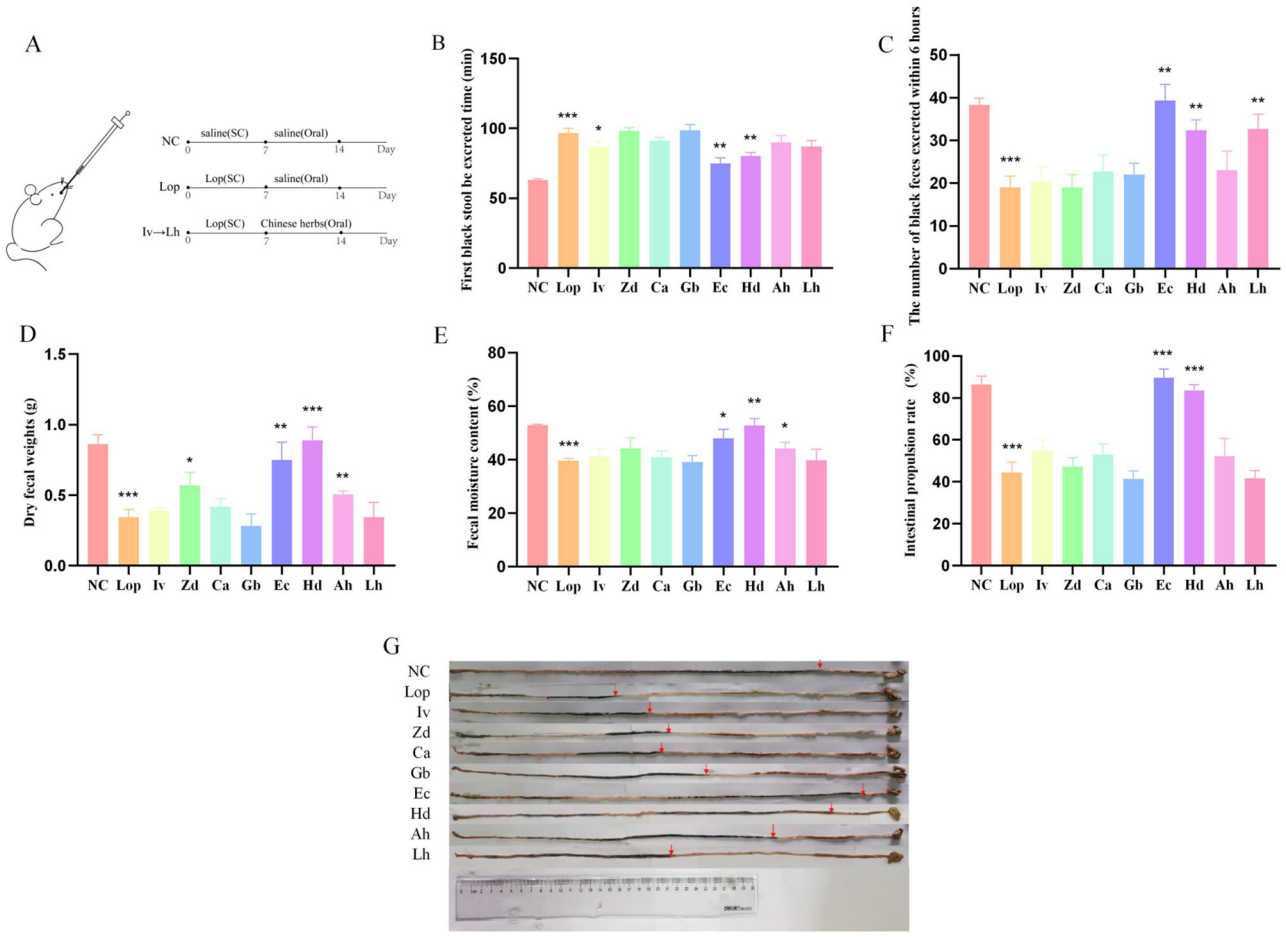
Effects of ECAE and HDAE on defecation parameters in a mouse model of constipation. **(A)** Animal model and treatment flow chart; **(B–F)** Fecal parameters. Compared with the NC group, ***P < 0.001; compared with the Lop group, *P < 0.05, **P < 0.01, ***P < 0.001; **(G)** Schematic representation of the intestinal propulsion rate. ECAE, *E. ciliata* aqueous extract; HDAE, *H. dulcis* aqueous extract; Lop, loperamide-treated; NC, normal control.

The overall experimental design is shown in Figure 1A. As shown in Figures 1B–G, compared with those in the normal control (NC) group, the number of black stool samples discharged from the loperamide-treated (Lop) group was significantly greater (*P* < 0.001), and the number of black feces discharged within 6 h was significantly lower (*P* < 0.001). Moreover, in the Lop group, the dry weight of the feces was decreased (*P* < 0.001), the fecal water content also tended to be decreased (*P* < 0.001), and the intestinal propulsion rate decreased accordingly compared with the NC group (*P* < 0.001). However, compared with those in the Lop group, the various fecal parameters significantly differed in the ECAE-treated (Ec) group and HDAE-treated (Hd) group. The time to the first black stool was significantly shorter (*P* < 0.01), the number of black feces excreted within 6 h was greater (*P* < 0.01), the dry weight and water content of the feces was higher (*P* < 0.05, *P* < 0.01 or *P* < 0.001), and the rate of intestinal propulsion was also significantly higher (*P* < 0.001) in the Ec and Hd groups than in the Lop group. These findings fully confirmed that ECAE and HDAE improved the defecation status and increased the intestinal motility of the mice in the constipation model.

### 3.2 ECAE and HDAE ameliorate the intestinal tissue structural and morphological damage caused by loperamide hydrochloride in mice

**Figure 2:**
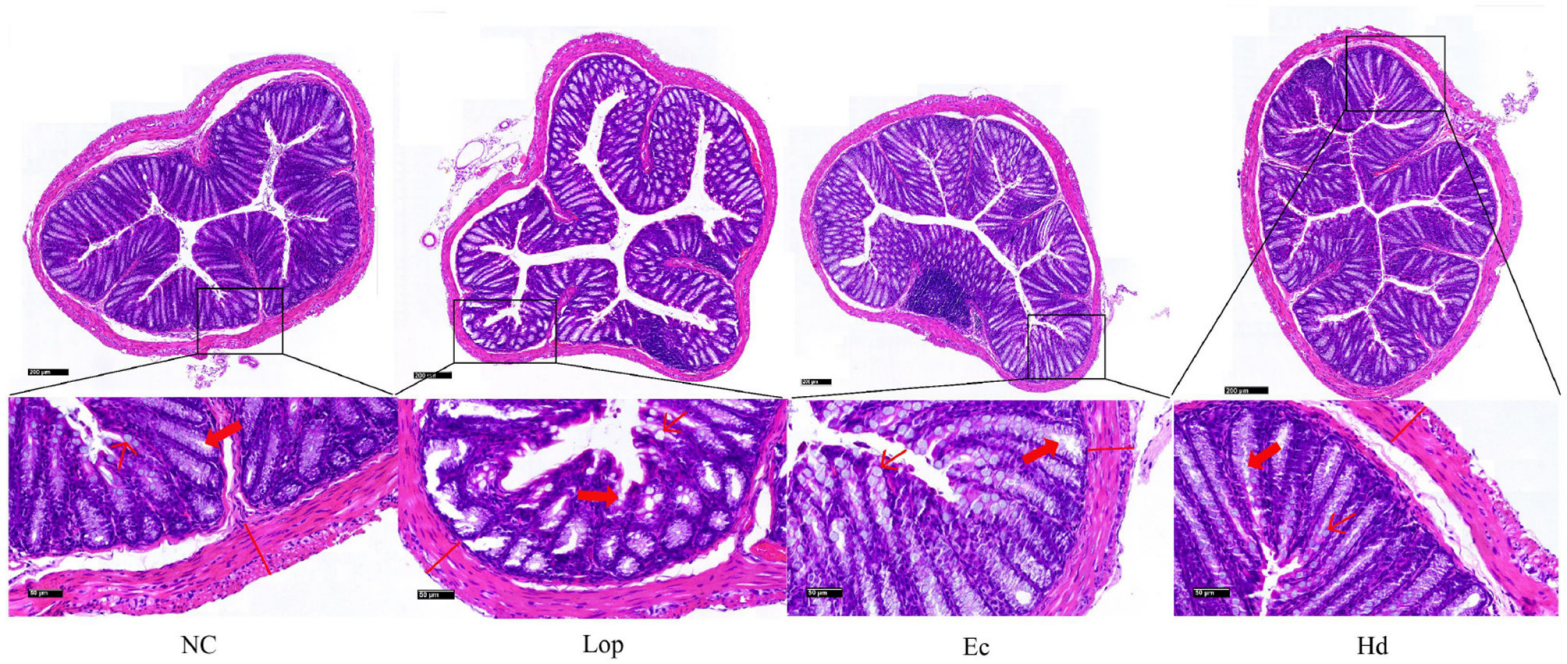
Effects of ECAE and HDAE on intestinal tissue structure and cell morphology induced by loperamide hydrochloride in mice. H&E staining was used to observe colon tissue sections from the NC group, the Lop group, the Ec group and the Hd group via light microscopy, with scale bars of 200 μm and 50 μm. 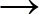, Goblet cells; 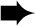, Intestinal crypts; 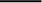, Muscularis externa. ECAE, *E. ciliata* aqueous extract; HDAE, *H. dulcis* aqueous extract; NC, normal control; Lop, loperamide-treated; Ec, ECAE-treated; Hd, HDAE-treated.

To determine the histological changes in the colons of Ec and Hd group mice, hematoxylin and eosin (H&E) staining was performed, as shown in Figure 2. The NC group presented a healthy and intact colonic histological structure with dense and well-arranged epithelial cells of the mucous layer and a separating layer between the mucosa and the submucosa, with morphologically normal goblet cells and intestinal crypts with regular surfaces. In contrast, the Lop group exhibited severe histological damage, with thinning of the muscularis propria, breakage and disappearance of the epithelial cells of the mucus layer, depletion and fracture of the goblet cells, atrophy and distortion of the crypt structure, and scattered inflammatory cell infiltration in the submucosa. However, the intestinal pathomorphological damage in the Ec and Hd groups was ameliorated, as indicated by intact and well-arranged epithelial cells in the mucous layer, increased numbers of goblet cells, and the restoration of the crypt structure to normal. These findings suggest that ECAE and HDAE can repair pathological damage to the colon in constipation model mice.

### 3.3 ECAE and HDAE increase the number of c-Kit- and SCF-positive cells in the colon of constipation model mice

**Figure 3:**
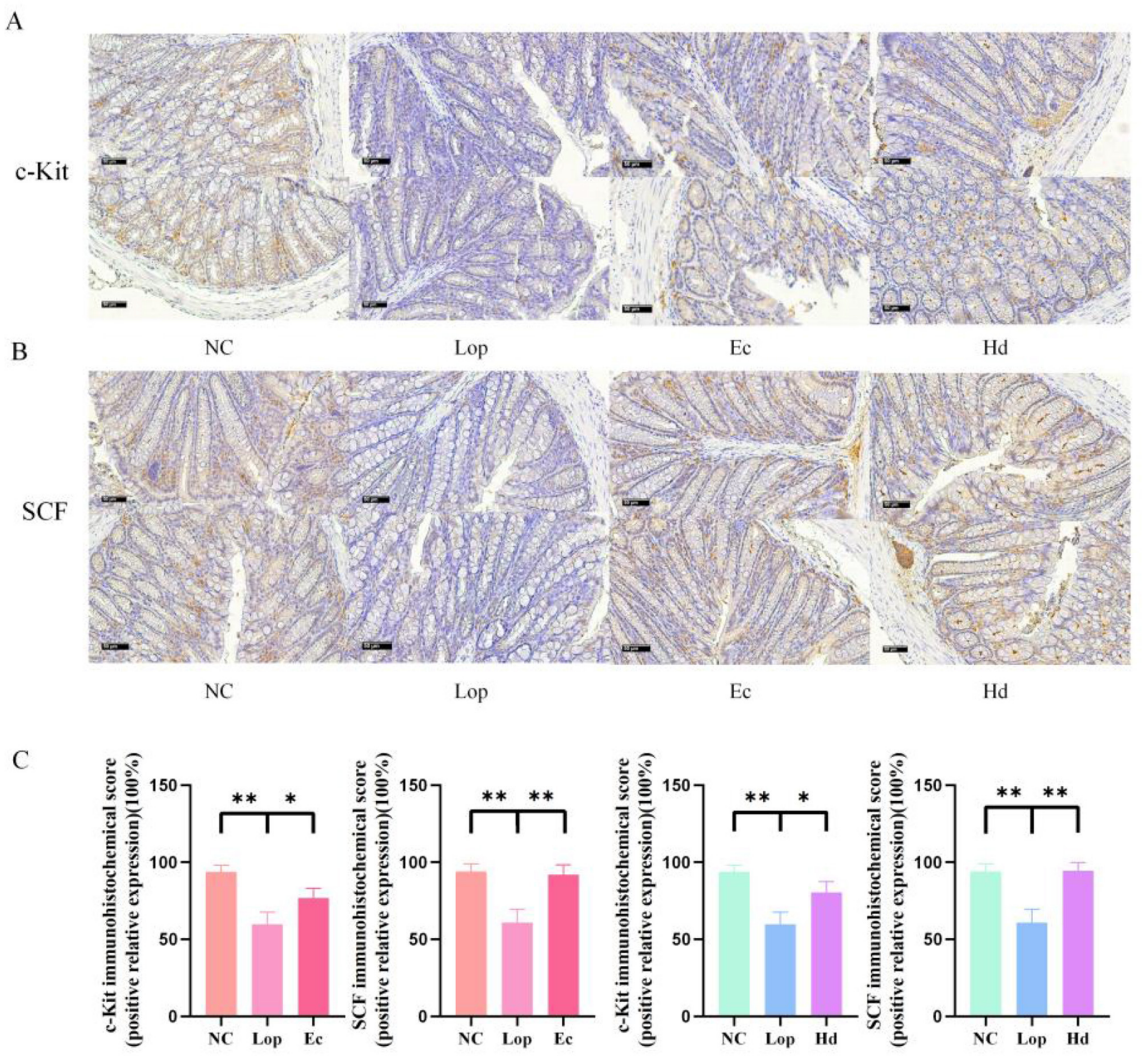
Regulation of c-Kit and SCF in the intestinal tissue of constipation model mice by ECAE and HDAE. **(A)** IHC images of c-Kit; **(B)** IHC images of SCF; Results of IHC were scored and expressed as shown in **(C)**. Compared with the NC group, **P < 0.01; compared with the Lop group, *P < 0.05, **P < 0.01. ECAE, *E. ciliata* aqueous extract; HDAE, *H. dulcis* aqueous extract; NC, normal control; Lop, loperamide-treated; Ec, ECAE-treated; Hd, HDAE-treated; IHC, immunohistochemistry; c-Kit, Stem Cell Factor Receptor; SCF, Stem Cell Factor.

The immunoreactivity of Stem Cell Factor Receptor (c-Kit) on the surface of interstitial cells of Cajal (ICCs) with its Stem Cell Factor (SCF) ligand was determined by immunohistochemistry. As shown in Figures 3A, B, analysis of the diaminobenzidine (DAB) staining results revealed that the expression of c-Kit and SCF in the intestinal tissues after loperamide treatment was significantly lower and sparsely distributed than that in the NC group. After treatment with ECAE or HDAE, the number of c-Kit- and SCF-positive cells significantly rebounded, and these cells were densely distributed, which was consistent with the results of the quantitative analysis (*P* < 0.05 or *P* < 0.01) as shown in Figure 3C. These findings suggest that loperamide treatment leads to a reduction in the number of or the dysfunction of ICCs in the mouse colon and that ECAE and HDAE may rescue this consequence by activating the c-Kit/SCF pathway, which in turn coordinates intestinal smooth muscle contraction and promotes intestinal peristalsis.

### 3.4 ECAE and HDAE increase the expression levels of ZO-1, Occludin and Claudin-1 in the colons of constipation model mice

**Figure 4:**
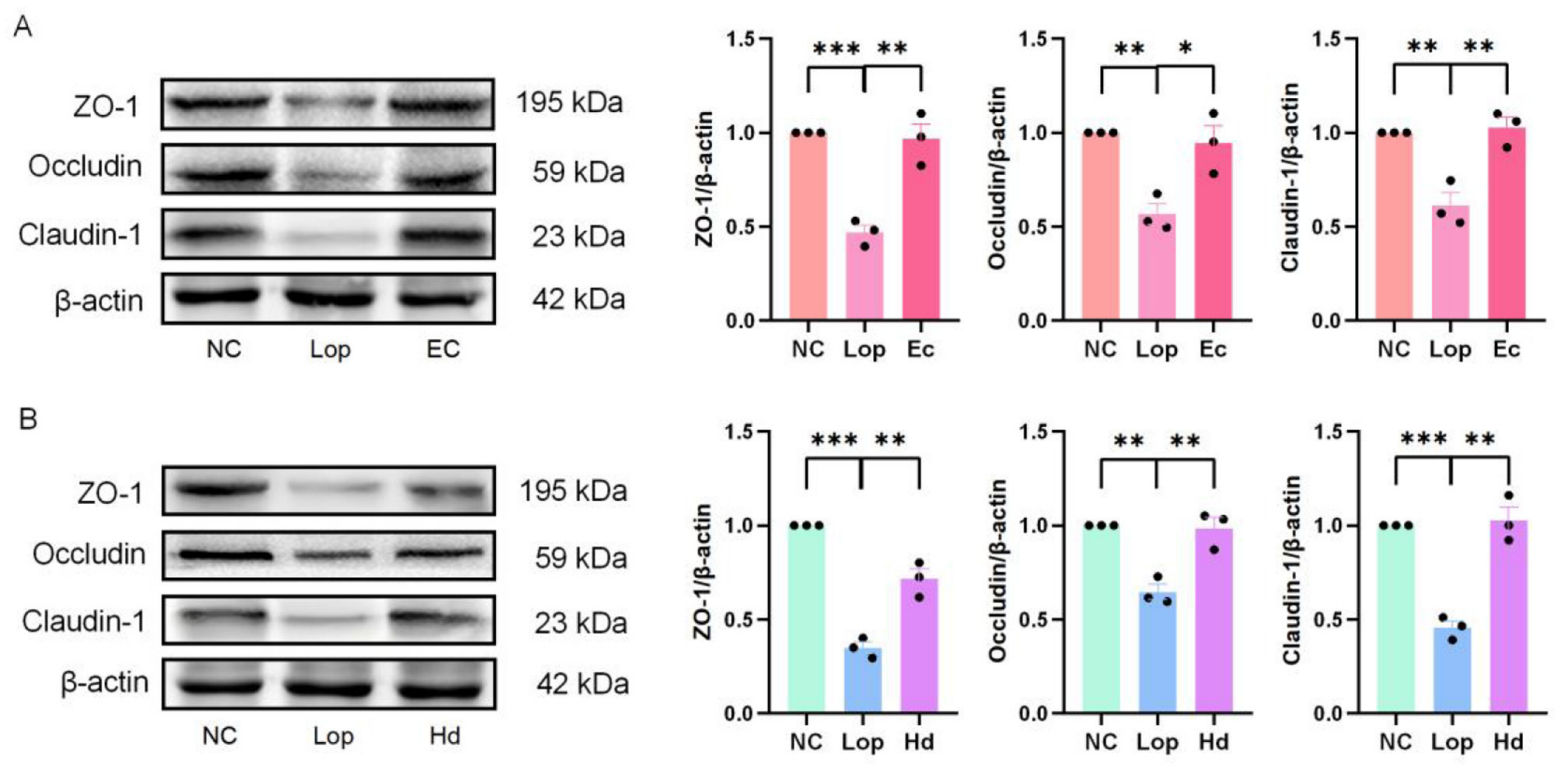
Effects of ECAE and HDAE on ZO-1, Occludin and Claudin-1 levels in the colons of constipation model mice. **(A, B)** Protein expression levels of ZO-1, Occludin and Claudin-1 were evaluated via western blot analysis, and the results of the quantitative analysis of gray intensity were calculated. Compared with the NC group, **P < 0.01, ***P < 0.001; compared with the Lop group, *P < 0.05, **P < 0.01. ECAE, *E. ciliata* aqueous extract; HDAE, *H. dulcis* aqueous extract; NC, normal control; Lop, loperamide-treated; Ec, ECAE-treated; Hd, HDAE-treated.

The authors further investigated the effects of ECAE and HDAE on the expression levels of three intestinal tight junction proteins, Zonula Occludens-1 (ZO-l), Occludin and Claudin-l, in the colonic tissues of constipation model mice. The immunoblotting results are shown in Figures 4A, B. Statistical analysis of the gray values of the bands revealed that the expression levels of tight junction proteins in the colons of mice in the Lop group were significantly lower than those in the colons of mice in the NC group (*P* < 0.01 or *P* < 0.001). However, the expression levels of tight junction proteins in the colons of the mice in the Ec and Hd groups were significantly greater (*P* < 0.05 or *P* < 0.01) than those in the colons of mice in the Lop group. These findings suggest that ECAE and HDAE can repair intestinal barrier damage in mice with loperamide-induced constipation.

### 3.5 Effects of ECAE and HDAE on the diversity of the intestinal flora in constipation model mice

**Figure 5:**
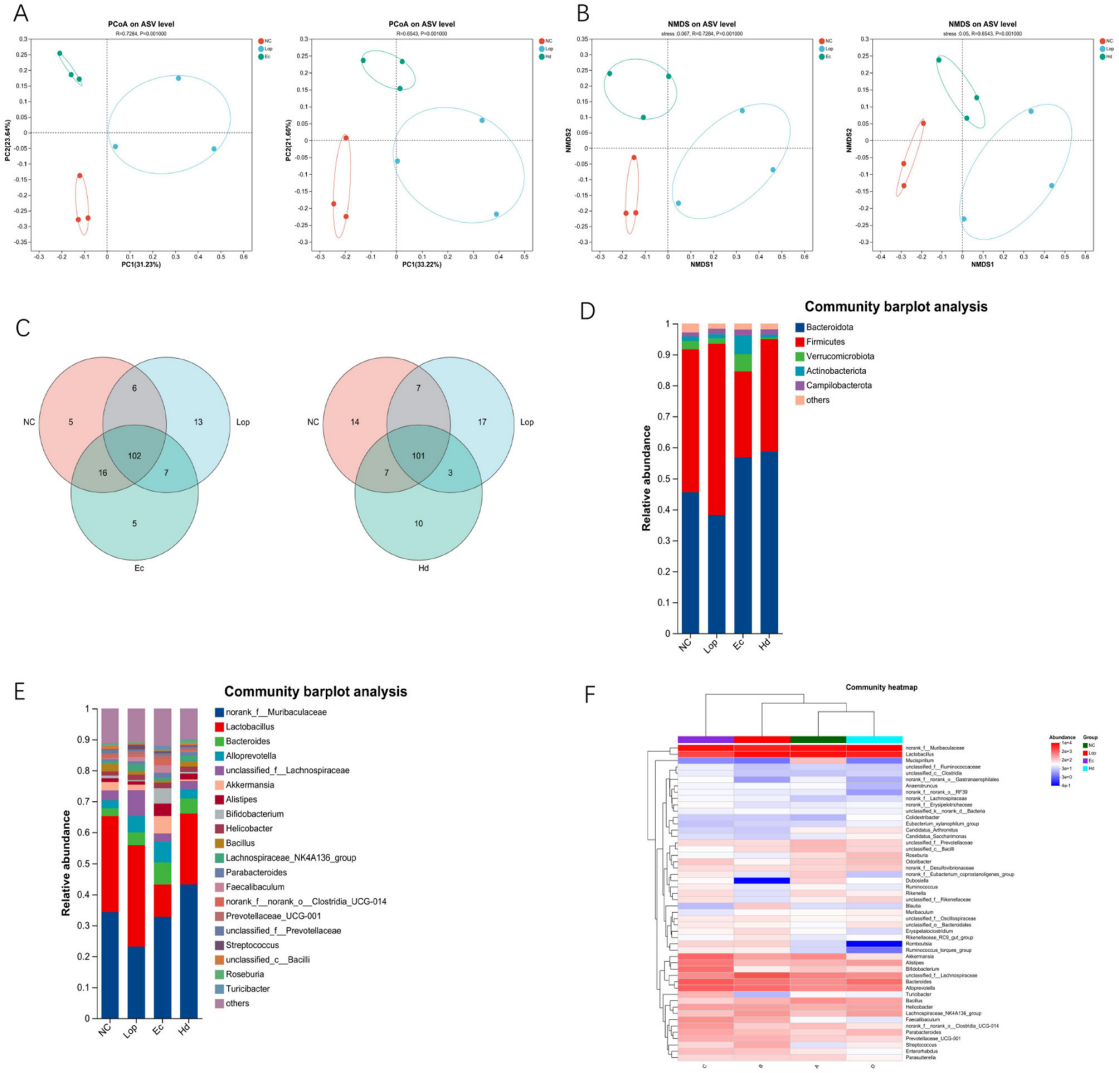
Effects of ECAE and HDAE on intestinal flora diversity in constipation model mice. **(A)** PCoA of the β diversity of the Ec and Hd groups; **(B)** NMDS of the β diversity of the Ec and Hd groups; **(C)** Venn diagram of bacterial sequences in the Ec and Hd groups compared with those in the NC and Lop groups; **(D)** Cluster bar map at the phylum level; **(E)** Cluster bar map at the genus level; **(F)** Cluster heatmap. ECAE, *E. ciliata* aqueous extract; HDAE, *H. dulcis* aqueous extract; NC, normal control; Lop, loperamide-treated; Ec, ECAE-treated; Hd, HDAE-treated; PCoA, principal component analysis; NMDS, nonmetric multidimensional scaling analysis.

The authors used 16S rRNA sequencing to detect changes in the intestinal flora composition in mice in the NC group, Lop group, Ec group and Hd group. To assess β diversity, the authors performed principal coordinate analysis (PCoA) and nonmetric multidimensional scaling analysis (NMDS) of the intestinal samples. The closer the sample points in the graph were, the more similar the species composition of the samples was. PCoA and NMDS revealed two significant clustering groups: the Ec group (pressure = 0.067) and the Hd group (pressure = 0.05; pressure < 0.2 indicates the validity of NMDS; Figures 5A, B). The results obtained from the β diversity analysis showed that the different treatments had significant effects on the intestinal flora of each group of mice. By comparing the sequences of the bacteria in the intestinal samples, the authors found that the microbiota compositions of both the Ec group and the Hd group were different from those of the NC and Lop groups. A total of 102 genera were common between the Ec group and the NC and Lop groups, and *g__Psychrobacter, g__Kocuria, g__unclassified_f__Lactobacillaceae, g__Rodentibacter*, and *g__Globicatella* were the species specific to the Ec group. A total of 101 genera were common between the Hd group and the NC and Lop groups, and *g__Eubacterium_ruminantium_group, g__norank_f__norank_o__Bacteroidales, g__Olsenella, g__Fusicatenibacter, g__Tyzzerella, g__Ruminiclostridium, g__norank_f__norank_o__Izemoplasmatales, g__unclassified_f__Marinifilaceae, g__Bilophila*, and *Ruminococcaceae_UCG-002* were species unique to the Hd group (Figure 5C). As shown in Figure 5D, at the phylum level, the relative abundances of Firmicutes and Bacteroidota were high in all groups. The ratio of *Firmicutes* to *Bacteroidota* in the Lop group was significantly lower than that in the NC group, and the ratios of *Firmicutes* to *Bacteroidota* in the Ec group and Hd group were significantly higher than that in the Lop group. As shown in Figure 5E, at the genus level, the relative abundances of *norank_f__Muribaculaceae* and *Lactobacillus* were high. Compared with that in the NC group, the ratio of *norank_f__Muribaculaceae* to *Lactobacillus* in the Lop group was significantly lower. Compared with that in the Lop group, the ratios of *norank_f__Muribaculaceae* to *Lactobacillus* in the Ec group and Hd group were significantly higher. The relative abundances of major bacterial genera in the NC, Lop, Ec and Hd groups are shown in heatmaps in Figure 5F. These results fully demonstrated that ECAE and HDAE could partially restore many aspects of loperamide-induced gut microbial dysbiosis, such as microbial diversity, community structure, and species composition.

### 3.6 Changes in the intestinal flora of constipation model mice after ECAE and HDAE treatment

**Figure 6:**
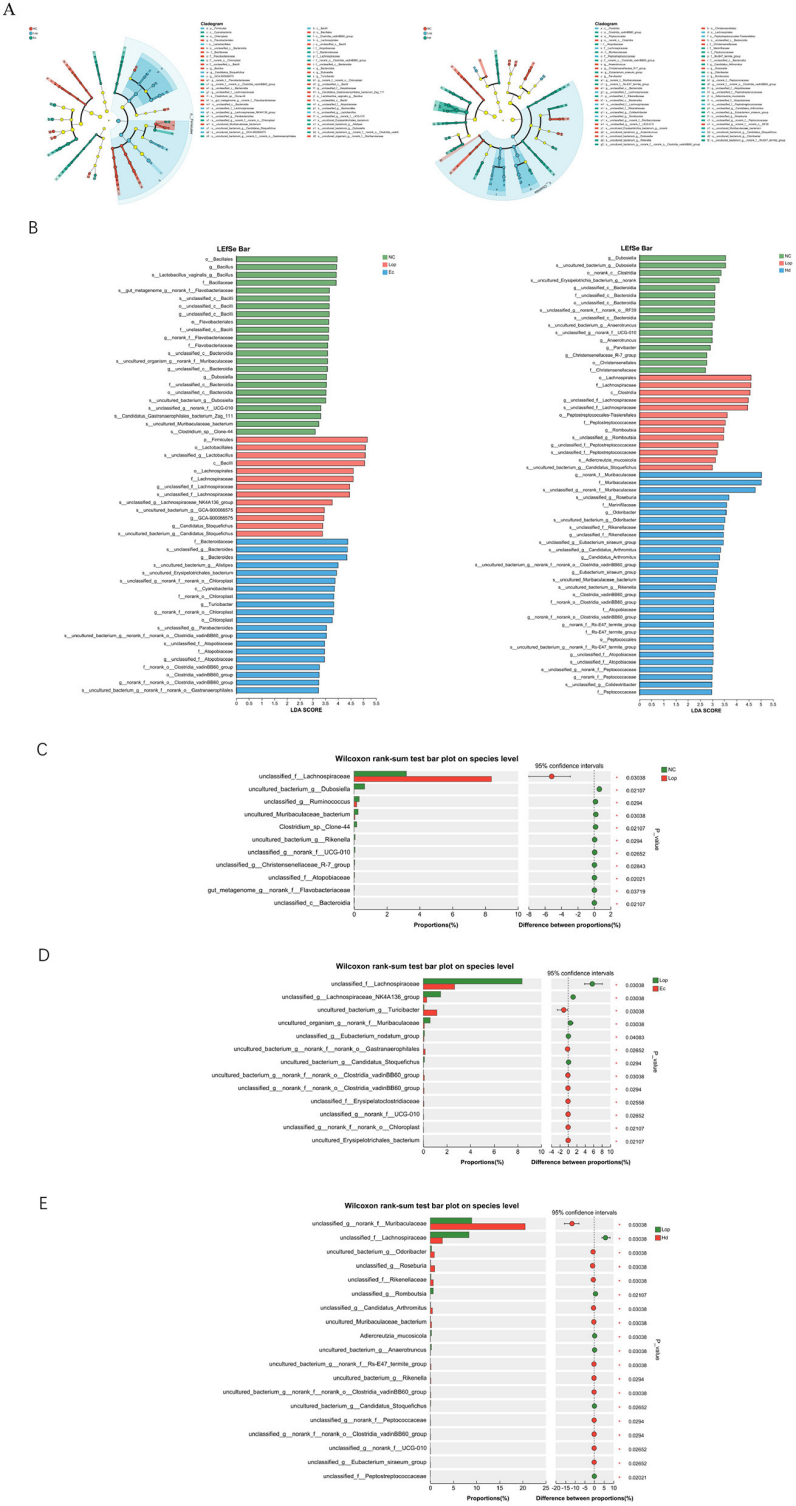
Changes in the intestinal flora in constipation model mice after ECAE and HDAE treatment. **(A)** Differences in species at different taxonomic levels presented as developmental dendrograms, which visually reflect the different species at different hierarchical levels among different subgroups; **(B)** LDA discriminant bar charts, with bars showing the LDA values of the various differential species, which visually demonstrate the magnitude of the influence of the unique species identified among different groups on the effect of the differences; the bars show the difference in the abundance of the same species among different groups in terms of the mean relative abundance differences between different groups, with labels indicating whether the differences are significant or not, visualizing the mean relative abundance differences of the same species between different groups. *P < 0.05. **(C)** NC group vs. Lop group; **(D)** Lop group vs. Ec group; **(E)** Lop group vs. Hd group. ECAE, *E. ciliata* aqueous extract; HDAE, *H. dulcis* aqueous extract; NC, normal control; Lop, loperamide-treated; Ec, ECAE-treated; Hd, HDAE-treated; LDA, linear discriminant analysis.

The authors used LEfSe (linear discriminant analysis effect size) analysis to identify biomarkers unique to the three groups at the phylum, order, order, family, genus, and species levels, setting the linear discriminant analysis (LDA) threshold at 2. As shown in Figures 6A, B, the dominant intestinal flora of the NC, Lop, and Ec groups were *o__Bacillales, p__Firmicutes, and f__Bacteroidaceae*, respectively. The dominant intestinal flora of the mice in the NC, Lop and Hd groups were *g__Dubosiella, o__Lachnospirales, and g__norank_f__Muribaculaceae*, respectively. After loperamide treatment and ECAE and HDAE treatment, the composition of the intestinal flora of the mice significantly changed. LEfSe multilevel species discrimination revealed that ECAE or HDAE treatment produced beneficial bacteria. The authors subsequently conducted a comparative analysis of the two groups, as shown in Figure 6C. Compared with the NC group, the abundances of *uncultured_bacterium_g__Dubosiella, unclassified_g__Ruminococcus, and uncultured_Muribaculaceae_bacterium* were significantly lower in in the intestinal flora of mice in the Lop group (*P* < 0.05). As shown in Figure 6D, compared with the Lop group, the abundances of *uncultured_bacterium_g__Turicibacter, unultured_bacterium_g__norank_f__norank_o__Gastranaerophilales, unultured_bacterium_g__norank_f__norank_o__Clostridia_vadinBB60_group, and unultured_Erysipelotrichales_bacterium* were significantly higher (*P* < 0.05), and the abundances of *uncultured_organism_g__norank_f__Muribaculaceae, unultured_bacterium_g__Candidatus_Stoquefichus* were significantly lower (*P* < 0.05), in the intestinal flora of the Ec group. As shown in Figure 6E, compared with the Lop group, the abundances of *uncultured_bacterium_g__Odoribacter, uncultured_Muribaculaceae_bacterium, uncultured_bacterium_g__norank_f__Rs-E47_termite_group, uncultured_bacterium_g__Rikenella*, and *uncultured_bacterium_g__norank_f__norank_o__Clostridia_vadinBB60_group* were significantly higher (*P* < 0.05), and the abundances of *Adlercreutzia_mucosicola, uncultured_bacterium_g__Anaerotruncus, and uncultured_bacterium_g__Candidatus_Stoquefichus* were significantly lower (*P* < 0.05), in the intestinal flora of Hd group mice. In summary, after treatment with ECAE and HDAE, the abundances of beneficial bacteria in the intestinal tract of constipation model mice increased significantly, which may have alleviated the symptoms of constipation by maintaining the balance of the intestinal flora, promoting the digestion and absorption of food, reducing the accumulation of harmful substances, and reducing the inflammatory response.

## 4 Discussion

Among eight ETCMs, ECAE and HDAE significantly ameliorated the symptoms of loperamide-induced constipation in ICR mice, ameliorated pathological tissue damage in the colons of constipation model mice, significantly increased the expression levels of intestinal peristalsis-associated proteins (c-kit and SCF) and intestinal barrier maintenance, significantly increased the expression levels of proteins related to the promotion of intestinal motility (c-Kit and SCF) and the maintenance of the intestinal barrier (ZO-1, Occludin and Claudin-1), and promoted the colonization of beneficial intestinal bacteria in constipation model mice.

Constipation is characterized by reduced intestinal peristalsis and difficulty defecating and is often accompanied by abdominal pain, bloating, and other discomfort (Black and Ford, 2018). The concept of ETCM underscores the commonalities of these symptoms; thus, the use of ETCMs may be an innovative approach for treating constipation. In this context, the authors conducted a literature review. They systematically examined 110 ETCMs recognized by the Ministry of Health as homologous to both medicine and food as of 2023 within the 2020 edition of the Chinese Pharmacopeia, among which 35 ETCMs, such as *Dioscorea oppositifolia* L., *Cannabis sativa* L., and *Amomum villosum Lour*., had primary therapeutic functions related to gastrointestinal health. These functions include tonifying the spleen and nourishing the stomach, moistening the intestines to alleviate laxity, and warming the spleen to mitigate diarrhea, among others. In modern research, the key words “constipation” and “laxative” were used to search the “Web of Science,” “PubMed,” “China Knowledge” and other scientific databases. The remaining 75 ETCMs were screened, and the roles of *I. verum, Z. dhumnades, C. aurantium, G. biloba, E. ciliata, H. dulcis, A. halys*, and *L. hypoglauca* in ameliorating constipation or promoting defecation have not been reported thus far. ETCM has emerged as a prominent research focus in numerous countries, highlighting the potential for managing diseases safely and effectively through dietary interventions rather than relying solely on pharmacological or surgical treatments. Research is being conducted on the efficacy of various ETCMs in the treatment of chronic conditions such as obesity, cancer, diabetes, and hypertension (Zheng et al., 2022; Liu Z. et al., 2023). By employing appropriate dietary combinations, some components can mimic the therapeutic effects of medications, thereby effectively controlling or even reversing disease progression. The evidence suggests that the Mediterranean diet offers significant cardiovascular protection and plays a crucial role in preventing and managing hypertension, hyperlipidemia, and related disorders (Wang et al., 2018). Additionally, a ketogenic diet has shown marked effectiveness in controlling seizures while also leading to positive outcomes in tumor treatment (Husari and Cervenka, 2021; Yang et al., 2024).

Fecal quantity, fecal water content and intestinal function are considered important factors in assessing the severity of constipation and drug efficacy (Vriesman et al., 2020). The authors initially assessed and documented the fecal parameters of experimental mice and discovered that ECAE and HDAE facilitated the expulsion of intestinal contents. The intestinal propulsion rates across all the groups were subsequently evaluated through mouse experiments, with visual data further substantiating that ECAE and HDAE improved defecation function by promoting intestinal peristalsis. To gain deeper insight into the impact of the herbal extracts on the intestinal microstructure, H&E staining was used to compare and analyze intestinal tissue sections obtained from NC, Lop, Ec and Hd group mice. The findings indicated that ECAE and HDAE treatment ameliorated damage to the intestinal mucosa and restored the structural integrity of intestinal layers to normal. This evidence may serve as a morphological basis for improving digestive and absorptive functions while alleviating constipation. These results align with previous reports indicating that certain drug extracts have therapeutic effects on constipation (Wang L. et al., 2023; Zhang et al., 2024).

In the gastrointestinal tract, Cajal interstitial cells (ICCs) constitute a distinct class of interstitial cells that form an intricate network between the enteric nervous system and smooth muscle (Fan et al., 2014). This network is essential for coordinating gastrointestinal motility and secretion. The c-Kit/SCF signaling pathway plays a pivotal role in this process by regulating ICC development, proliferation, and survival, thereby ensuring the proper functioning of the network (Satoshi et al., 2020). Immunohistochemical protein expression assays revealed that ECAE and HDAE regulated the expression levels of proteins closely related to intestinal peristalsis, such as SCF and c-Kit, which explains, at the molecular level, how these two aqueous extracts can regulate intestinal motility and improve intestinal function. *Prunus persica* (L.) Batsch blossom soluble dietary fiber synergia polyphenol was demonstrated to increase the protein and mRNA expressions of SCF and c-Kit in the c-Kit/SCF signaling pathway to speed up gut movement (Liang et al., 2024). *Suaeda salsa* (L.) Pall was found to upregulate c-Kit and SCF protein levels, promote the proliferation of the main intestinal rhythm generators, ICCs, and thus increase intestinal peristalsis (Zhang et al., 2024). Furthermore, the efficacy of constipation treatment can be inhibited by blocking the SCF/c-Kit signaling pathways (Zhang et al., 2021).

Alterations in mouse intestinal pathological morphology reflect the importance of intestinal mucosal barrier function in the treatment of constipation. Short-chain fatty acids (SCFAs) play a crucial role in maintaining the integrity of tight junctions between intestinal epithelial cells (Guo et al., 2021). For instance, butyric acid, as a representative SCFA, enhances the mechanical function of the intestinal mucosal barrier by upregulating the expression of ZO-1 and Occludin, and by modulating the distribution of Claudin-1 on the cell surface (Soret et al., 2010; Rekha et al., 2024). Western blot analysis provided quantitative evidence for the specific effects of ECAE and HDAE on the expression levels of the intestinal proteins ZO-1, Occludin, and Claudin-1, confirming their positive roles in protecting the mucosal barrier. This insight offers a novel approach to investigating the intestinal microbiota in constipated mice, particularly focusing on butyrate-producing bacteria. *Cymbopogon citratus* (DC.) Stapf aqueous extract has been shown to restore loperamide-induced impaired barrier function and maintain intestinal homeostasis by increasing the mRNA expression levels of mechanical barrier proteins (Occludin and ZO-1; Gao et al., 2022). The water extract of *Cannabis sativa* L. was found to significantly reverse the reduction in the expression of gut barrier-related molecules, including Claudin-1 and Occludin, at the mRNA level in constipation model mice (Li et al., 2022).

The ratio of Firmicutes to Bacteroidetes is an important indicator of the balance of the intestinal flora (Wu et al., 2021). 16S rRNA sequencing revealed that, at the phylum level, Firmicutes and Bacteroidetes were the dominant intestinal flora in all groups of mice. Consistent with the authors' study, a decrease in the proportion of Firmicutes and an increase in the proportion of Bacteroidetes was observed in patients with functional constipation (Hu et al., 2019). ECAE and HDAE reversed the decrease in the ratio of Firmicutes to Bacteroidetes caused by loperamide, increased the proportion of Firmicutes, and restored the imbalanced structure of the intestinal flora. Many bacteria within the Firmicutes phylum are beneficial, such as Lactobacillus, which not only promotes intestinal peristaltic secretion but also inhibits the growth of harmful bacteria. This action helps restore the balance of the intestinal flora and alleviates constipation. Additionally, Firmicutes are the primary producers of butyrate, a compound that plays a crucial role in the gastrointestinal tract. As an important SCFA, butyrate plays a crucial role in maintaining intestinal microecological balance. Butyrate is particularly important for repairing damaged intestinal wall cells, improving intestinal barrier function (Zheng et al., 2017), alleviating the intestinal inflammatory response (Lee et al., 2016) and alleviating constipation symptoms by stimulating water and electrolyte absorption in the colon, thereby reducing stool volume. More importantly, butyric bacteria and probiotics that colonize the intestine have synergistic effects on the growth of harmful bacteria (Ye et al., 2018). *Paeniclostridium, Coriobacteriales, Macrococcus, Faecalitalea* and other pathogenic bacteria disappeared from the endemic flora of the Lop group after ECAE treatment. Compared with those in the Lop group, the abundances of butyrate-producing bacteria such as *Turicibacter* (Zeng et al., 2024)*, Clostridia_vadinBB60_group* (Huang et al., 2024), and *Erysipelotrichales* (Li et al., 2020) in the Ec group were significantly higher. After HDAE treatment, *Enterococcus, Coriobacteriales, Peptostreptococcales-Tissierellales, Paeniclostridium, Macrococcus, Faecalitalea* and other pathogenic bacteria from the Lop group of the unique bacterial groups disappeared from the Lop group. Compared with the Lop group, the Hd group exhibited new butyrate-producing bacteria, including *Olsenella* (Shi et al., 2022)*, Fusicatenibacter* (Zeng et al., 2024)*, Ruminiclostridium* (Liu et al., 2021)*, Tyzzerella* (Xu et al., 2022), and *Ruminococcaceae_UCG-002* (Liao et al., 2021), after treatment. HDAE promoted the growth of butyrate-producing bacteria of *Odoribacter* (Liu et al., 2021)*, Muribaculaceae* (Liu P. et al., 2023)*, Rikenella* (Shi et al., 2017)*, Clostridia_vadinBB60_group* (Huang et al., 2024)*, etc*. Studies have shown that some Chinese herbs can play a “prebiotic” role after entering the intestine, enriching beneficial butyrate-producing bacteria. For example, a water insoluble polysaccharide (WIP) was isolated and identified from *Poria cocos*, and the abundances of the mouse cecal butyrate-producing bacteria *Lachnospiracea* and *Clostridium* increased after treatment (Sun et al., 2019). Gegen Qinlian Decoction (GQD) is a TCM formula that enriches the rat gut with many butyrate-producing bacteria, including *Faecalibacterium* and *Roseburia*, thereby attenuating intestinal inflammation and lowering glucose (Xu et al., 2020). In summary, ECAE and HDAE can increase the diversity of the intestinal flora, increase the proportion of beneficial bacteria, reduce the proportion of harmful bacteria, and improve the intestinal microecological environment, all of which may be important ways to alleviate constipation.

The limitations of this study include the lack of determination of the optimal dose and route of administration for ECAE and HDAE, which currently precludes an assessment of their effective application in clinical settings. Consequently, in future research, multiple dosing groups and various routes of administration should be established on the basis of the findings from this study to ascertain the optimal dosing and administration parameters for ECAE and HDAE. Additionally, subsequent investigations could be focused on refining the isolation and purification processes for the active ingredients in ECAE and HDAE, on validating the critical role of specific intestinal flora in constipation development through Fecal Microbiota Transplantation (FMT), providing a theoretical foundation for personalized extract treatments, as well as on evaluating the mechanisms of action and clinical effects of ECAE and HDAE in humans.

In this study, for the first time, evidence is presented regarding the laxative effects and mechanisms of action associated with non-beneficial gastrointestinal function related to ETCMs. The results indicate that ECAE and HDAE have significant potential in treating constipation by increasing intestinal propulsion rates, reducing defecation times, improving the intestinal mucosal structure, regulating the expression of proteins linked to intestinal barrier integrity, and optimizing the composition of the gut microbiota. This research transcends traditional pharmaceutical or surgical treatment modalities by laying a foundation for integrating dietary modifications with lifestyle changes; such an approach may provide a safer and more effective treatment strategy for patients suffering from constipation.

## Data Availability

The data presented in the study are deposited in the NCBI repository, the accession number(s) is PRJNA1256912.
